# E-prescribing and access to prescription medicines during lockdown: experience of patients in Aotearoa/New Zealand

**DOI:** 10.1186/s12875-021-01490-0

**Published:** 2021-07-01

**Authors:** Fiona Imlach, Eileen McKinlay, Jonathan Kennedy, Caroline Morris, Megan Pledger, Jacqueline Cumming, Karen McBride-Henry

**Affiliations:** 1 Health Services Research Centre, Victoria University of Wellington, P.O. Box 600, Wellington, 6011 New Zealand; 2 Department of Primary Health Care and General Practice, University of Otago Wellington, PO Box 7343, Newtown, 6021 New Zealand; 3 School of Nursing, Midwifery, and Health Practice, Victoria University of Wellington, P.O. Box 600, Wellington, 6011 New Zealand

**Keywords:** Prescriptions, General practice, Primary health care, Community pharmacies, Coronavirus, Pandemics, Lockdown

## Abstract

**Background:**

Health services internationally have been compelled to change their methods of service delivery in response to the global COVID-19 pandemic, to mitigate the spread of infection amongst health professionals and patients. In Aotearoa/New Zealand, widespread electronic delivery of prescriptions (e-prescribing) was enabled. The aim of the research was to explore patients’ experiences of how lockdown, changes to prescribing and the interface between general practices and community pharmacy affected access to prescription medications.

**Method:**

The research employed a mixed-method approach. This included an online survey (*n* = 1,010) and in-depth interviews with a subset of survey respondents (*n* = 38) during the first COVID-19 lockdown (March–May 2020). Respondents were recruited through a snowballing approach, starting with social media and email list contacts of the research team. In keeping with the approach, descriptive statistics of survey data and thematic analysis of qualitative interview and open-ended questions in survey data were combined.

**Results:**

For most respondents who received a prescription during lockdown, this was sent directly to the pharmacy. Most people picked up their medication from the pharmacy; home delivery of medication was rare (4%). Survey and interview respondents wanted e-prescribing to continue post-lockdown and described where things worked well and where they encountered delays in the process of acquiring prescription medication.

**Conclusions:**

E-prescribing has the potential to improve access to prescription medication and is convenient for patients. The increase in e-prescribing during lockdown highlighted how the system could be improved, through better feedback about errors, more consistency across practices and pharmacies, more proactive communication with patients, and equitable prescribing costs.

**Supplementary Information:**

The online version contains supplementary material available at 10.1186/s12875-021-01490-0.

## Background

Before the COVID-19 pandemic and lockdown events, electronic or e-prescribing (with modified or reduced need for physical signatures on prescriptions in Aotearoa/New Zealand (A/NZ)) was only available to general practices using the New Zealand electronic Prescribing System (NZePS). During the Stay-at-home’ lockdown in March/April 2020, (when only supermarkets, pharmacies and health services were open), to reduce use of paper scripts as a possible vector for COVID-19 transmission, uptake of the NZePS increased from 30 to 80% of general practices and the Government introduced waivers to allow other health services (e.g. hospital discharge and outpatient clinics) to use e-prescribing.

The global COVID-19 pandemic has compelled health services to change how they deliver health care, as it has infected millions of people worldwide, caused over a million deaths [1], and is easily spread, mostly through respiratory secretions or droplets but also through contact with contaminated surfaces [2]. Hence, both health professionals and patients, particularly those who are older or have co-morbidities, are at risk of infection.

In A/NZ, the need to protect individuals, reduce transmission of the virus and maintain a functioning health system were at the heart of changes to health service delivery [3]. Similar to other countries, services that previously delivered all or most of their care in-person shifted to remote ways of working, such as telephone and video consultations (telehealth) [4–6]. Electronic methods of prescribing (e-prescribing) were introduced or accelerated, to minimise contact between health professionals, pharmacists and patients [7, 8]. The rapid shift to telehealth and e-prescribing was prompted by the Government’s decision to put the country into ‘lockdown’ in response to the COVID-19 pandemic (see Table 1).

**Table 1:** Summary of Aotearoa/New Zealand (NZ)’s initial COVID-19 response

28/02/2020	First recognized case of COVID-19 in NZ (although an earlier historical case was subsequently detected)
11/03/2020	Declaration of a pandemic by the World Health Organization
16/03/2020	The New Zealand Government required all visitors to the country to self-isolate for 14 days; public gatherings of more than 500 people restricted
19/03/2020	Borders and entry ports were closed to all non-residents (with some exceptions); indoor gatherings of more than 100 people restricted
21/03/2020	A newly minted four-level Alert system was introduced, to guide how individuals, businesses and the nation would operate at different levels of pandemic threat [9]; NZ set at Alert Level 2
23/03/2020	Moved up to Alert Level 3 (equivalent to a partial lockdown), schools closed, some other businesses were able to open, as long as there was no physical contact with customers; health services were to be delivered remotely as much as possible
25/03/2020	Moved up to Alert Level 4 (equivalent to a complete nationwide lockdown) during which people were told to stay at home and only essential businesses remained open, which included specified health services and community pharmacies. These were instructed to implement recognised COVID-19 infection control measures, which included hand washing/sanitising, physical distancing, replacing in-person visits with telehealth where possible, and cessation of some routine services [10]
27/04/2020	Moved down to Alert Level 3, allowing gatherings of 10 people, early childhood centres and some schools reopened
13/05/2020	Moved down to Alert Level 2, which maintained physical distancing and limits on size of gatherings but allowed all businesses and schools to re-open

Before lockdown, only hard-copy prescriptions that were personally signed by a clinician were legal under the Medicines Act 1981; these prescriptions were taken to a pharmacy by an individual or sent by fax or post [11]. The only exception was for general practices using the secure New Zealand ePrescription Service (NZePS), which includes an enduring waiver for signature-less prescriptions [12]. During lockdown, uptake of the NZePS increased from around 300 general practices at the end of 2019 to 800 by April 2020 [12], from a total of around 1000 practices.

For practices and other parts of the health system without access to NZePS (e.g. hospital discharge and outpatient clinics), the Ministry of Health put in place a temporary waiver to the Medicines Act to allow prescriptions to be sent to community pharmacies through secure email systems (see Table 2) [13]. This widening of the legislative waiver encouraged contactless transmission of prescriptions, and accommodated clinicians working from home without access to a printer, fax or scanner. Similar legislative changes to support e-prescribing in response to COVID-19 were also implemented in other countries [14].

**Table 2:** Signature exempt e-prescribing during Covid-19 [7]

During Covid-19 it was possible to send e-prescriptions to a pharmacy in the two ways described below. The patient did not necessarily require a consultation with the prescriber before an e-prescription was issued; repeat prescriptions could also be processed in this way. However generated, all signature exempt prescriptions are directly linked back to the prescriber by the presence of the prescriber’s name and their New Zealand Medical Council number on the prescription
***Using the New Zealand electronic Prescribing System (NZePS)***
NZePS is a secure messaging channel that enables prescriptions to be generated and transmitted to a pharmacy electronically, similar to systems in the United Kingdom (UK) [15, 16] and Australia [17]. Using NZePS is expected to improve communication between practices and pharmacies, allow the prescriber to see the status of the prescription (dispensed, cancelled, repeats remaining) and also what has previously been prescribed [18]. All community pharmacies have had access to the NZePS system since 2016 but not all general practices, as access was only available through some (albeit commonly used) practice management systems (PMSs). In addition, activation incurred an additional cost to some practices, depending on their PMS [18]
***Without NZePS***
During Covid-19 a temporary waiver was issued by the Director General of Health to enable non-NZePS signature exempt prescriptions providing certain criteria are met, including transmission using a defined “secure” electronic system and that the prescription is in a form that is hard to change for anyone other than the original prescriber (e.g., a PDF or photograph). This waiver has recently been extended to 21^st^ June 2021 [13]

The other major change to prescribing during lockdown was to restrict dispensing to only 1 month of medicines at a time (previously 3 months for non-restricted government-funded medicines) [19]. This was also done in Canada, although both countries were criticised for the potential for this to cause significant stress to patients [20], despite the justification being to avoid drug shortages from global supply chain delays and patient stockpiling.

There is limited research available on e-prescribing and patients’ perspectives. Patient-focused research has indicated that pharmacy customers are largely satisfied with e-prescription systems, with convenience being the primary reason for this. However, patients have been reliant on information being provided by a health care professional to understand how the system worked [21–23].

Researchers have highlighted that e-prescribing supports safe prescribing due to reductions in prescription error in some instances [24]; however, e-prescribing policies have often lagged behind what practice [25]; an issue that has hampered efforts to introduce e-prescribing during the COVID-19 pandemic [26–28]. During the panedmic e-prescribing has been introduced in countries that had not previously used this approach, resulting in pharmacists calling for an extended scope of practice to better support the communities they work with [14]. The majority of research exploring e-prescribing during the pandemic is contained within research and commentary on telemedicine and does not address e-prescribing directly [21–23, 26–31].

The overall aims of this research were to gain patients’ perspectives on the changes to health service delivery during lockdown, how these changes affected access to care and what changes should persist post-lockdown. Given the mixed-method approach adopted for this research, the amount of data generated was considerable and publishing several pieces of distinct research enabled the findings to be explored in-depth. Other publications report on patients’ experiences of telehealth in general practice [32] and delays in accessing health care during lockdown [33]. In this article, we focus on the impact of changes to prescribing during lockdown. Research questions were:

- What were patients’ experiences of accessing prescriptions during lockdown?
- How did lockdown and changes to health service delivery affect their ability to obtain prescription medicines?
- What prescribing services would patients like to have in the future?

(See Table 3 for background information on primary health care and community pharmacy in NZ.)

**Table 3:** Primary health care and community pharmacy in Aotearoa/New Zealand

Primary health care in NZ is predominantly delivered through general practices, which act as gatekeepers to specialist services. Most people enrol in a general practice, which then receives a weighted capitation payment per quarter from the Government, with higher amounts given for enrolled populations with greater deprivation and health need [34]. General practices primarily operate as small businesses and charge co-payments for services. In addition to medical prescribers, several other health professional groups, including nurse practitioners, dentists, some nurses and a small number of pharmacists, can prescribe, usually within a specific scope of practice. Most medicines are subsidized but a co-payment of $5 per dispensed item is charged for the first 20 items per individual or family per year (Medicines Act 1981). Prescriptions and standard consultations to general practices are free for children under 14 years of age.
Community pharmacies, which are either small businesses or franchised chains, are variously located: some are co-located inside or next to general practices; others are inside shopping malls or large supermarkets; others are stand-alone in community shopping areas. Some online services with home delivery of medication have also developed recently. NZ law requires a pharmacist to be on-site at all times to dispense or check the dispensing of other technician staff and to advise customers on medicines. Patients choose which pharmacy to use and are not required to use the same pharmacy for every prescription

## Methods

This project took a mixed-methods approach, using both quantitative and qualitative analyses to enhance understanding of the topic [35–37]. We conducted a convenience sample survey (*n* = 1,010 responses), which included closed and open-ended questions. The survey was online from 20 April to 13 May 2020, which included the ‘Stay-at-home’ lockdown. Survey respondents were recruited through snowballing, starting with social media and email list contacts of the researchers, with the target group being those who used or wanted to use primary health care services during lockdown. In-depth interviews were then conducted with 38 survey respondents. These were selected from a subset of 436 respondents who volunteered to be followed up by providing contact details at the end of the survey. From these, we purposefully sampled by gender and sent invitations to 75 people, of whom 38 completed an interview (others did not respond; three could not complete the interview within the project timeframe). Interviewees were emailed an information sheet and consent form and gave oral or written consent prior to the interview. Most interviews were done via Zoom video-conferencing software, with four conducted by telephone. All were audio-recorded and transcribed, and interviewees had the opportunity to review transcripts. Interviews took place from 4–28 May 2020; more than half during the ‘Stay-at-home’ lockdown period of Alert Levels 3 and 4, the remainder while in Level 2 (see Table 1) [9].

We analysed closed-ended survey questions about how respondents got their prescriptions and prescription medicines, and preferences for prescribing services in the future, with descriptive statistics. In-depth interviews and open-ended survey responses (particularly for the question: ‘is there anything else you want to tell us about health care during lockdown’) were initially analysed separately by FI and KMH using NVivo 12. Themes around barriers and facilitators to accessing prescription medication were identified. This analysis was reviewed and checked for interpretation by the whole team to ensure there was agreement about participants’ experiences; these phases were consistent with the thematic analysis process described by Braun et al. [38] and led to further refinement of the themes. The final analysis presented here was the result of an iterative dialogical between members of the multi-disciplinary research team, including a pharmacist (CM) and general practitioner (JK), during which the different aspects of the findings were woven together to produce a coherent mixed-method analysis [36]. Quotes to demonstrate themes are inserted verbatim, indicating the age range and gender of the respondent and whether from the survey (S) or interviews (I).

## Results

It was not necessarily apparent to the patient how non-paper prescriptions were sent to the pharmacy. We therefore use the term ‘e-prescription’ to refer to any type of prescription that is computer generated by the clinician (in response to an online, telephone or in-person request) and conveyed to the pharmacy by electronic means, including fax, secure email or NZePS.

### Sample characteristics

Characteristics of survey respondents and interviewees are shown in Table 4. More detail about the sample can be found elsewhere [32]. There were more females and people from the lower North Island of NZ in the survey than expected. Interviewees were more likely to be older and not in employment or looking for work than survey respondents, likely because these groups had more time and willingness to be interviewed.

**Table 4:** Demographics of survey respondents and interviewees

**Characteristic**	**Survey** **n (%)** **Total *n* = 1,010**	**Interviewees** **n (%)** **Total *n* = 38**
**Age**		
18–34	221 (22)	7 (18)
35–44	201 (20)	6 (16)
45–54	247 (25)	12 (32)
55–64	173 (18)	3 (8)
65 +	145 (15)	10 (26)
**Gender**		
Female	840 (85)	24 (63)
Male	141 (14)	14 (37)
Other^a^	13 (1)	-
**Prioritised ethnicity (in order of priority)**		
Māori	101 (10)	6 (16)
Pacific peoples	18 (2)	3 (8)
Asian	34 (3)	4 (11)
New Zealand European/Other	833 (85)	25 (66)
**Current work status**		
In paid employment as before COVID-19	581 (59)	22 (58)
In paid employment with reduced pay due to COVID-19	108 (11)	3 (8)
In paid employment but not being paid due to COVID-19	26 (3)	–
Unemployed and looking for a job	31 (3)	–
Not in paid employment and not looking for a job	240 (24)	13 (34)
**Grouped District Health Board (DHB) areas**^b^		
Upper North Island	205 (21)	7 (18)
Central North Island	118 (12)	3 (8)
Lower North Island	437 (44)	20 (53)
South Island	232 (23)	8 (21)

^a^Those who answered “gender diverse” or “prefer not to say” were grouped together because of small numbers

^b^Upper North Island = Northland, Waitematā, Auckland and Counties Manukau DHBs; Central North Island = Waikato, Bay of Plenty, Tairāwhiti, Lakes, Taranaki DHBs; Lower North Island = Whanganui, Hawke’s Bay, MidCentral, Wairarapa, Hutt, Capital and Coast DHBs; South Island = Nelson-Marlborough, West Coast, Canterbury, South Canterbury, Southern DHBs

### Survey results

Eighty-six percent of respondents had contact with general practice during lockdown, including by phone, email or through an online patient portal. Of these, 74% obtained a prescription (either a repeat or new prescription). Just under half (48%) of these were from a consultation with a doctor or nurse (either telehealth or in-person consultation); otherwise, prescriptions were obtained by phoning the practice (23%) or ordering online (through an online general practice portal, email or website) (20%). The remaining were not clearly specified or obtained by other means.

During lockdown, in most cases, the patient (or their representative) collected the medicine from the pharmacy after the general practice had sent an e-prescription (81%). In an additional 4% of cases, the pharmacy sent the medicine to the patient’s home (after receiving the e-prescription from the practice). Only 14% of patients (or their representative) took a physical prescription to the pharmacy and then collected the medicine; fewer than 1% of respondents reported not filling the prescription (e.g. medicine out of stock, not yet needed or still waiting) (See Table 5).

**Table 5:** Specific questions asked in the survey from respondents who got a prescription during lockdown and how they got their medicine

	**n**	**%**
**Have you got a prescription from general practice during lockdown? (Total = 866)**		
Yes	637	74
No	229	26
**How did you get the prescription? (Total = 636)**		
Got it from a consult with the doctor or nurse	303	48
Phoned and left a message or spoke to someone at the clinic	147	23
Ordered it online (through a portal, email or website)	130	20
Picked it up from the clinic	23	4
Repeat prescription	2	0
Not specified / other	31	5
**How did you pick up the medicine? (Total = 633)**		
The clinic sent the prescription to a pharmacy and I (or someone on my behalf) collected the medicine(s)	510	81
I (or someone on my behalf) took the prescription to a pharmacy and collected the medicine(s)	88	14
The clinic sent the prescription to a pharmacy and the pharmacy sent the medicine(s) to me	26	4
Not filled (e.g. still waiting, not yet needed, medicine out of stock)	3	0
Other (e.g. dispensed at the clinic)	6	1

Most people who contacted general practices during lockdown were aware that before lockdown, practices could electronically send a prescription to the pharmacy (87%) or that they could order a prescription online (68%). Amongst all survey respondents, including those who may receive prescriptions via specialist/other services, the overwhelming majority wanted these types of services to continue post-lockdown: 91% wanted prescriptions sent electronically to the pharmacy and 89% wanted to order them from the general practice online (See Table 6).

**Table 6:** Awareness of services and what patients want in the future

	**n**	**%**
**Were you aware of any of the following services at the GP clinic before 23 March 2020 (before the coronavirus pandemic)?**^**a**^** (Total = 866)**		
Having prescriptions faxed to your pharmacist (so you don’t need to go into the clinic to pick up the prescription)	752	87
Ordering prescriptions online	588	68
**Which of the following services would you like your GP clinic/health centre to offer in the future (once the coronavirus pandemic is over)?**^**b**^** (Total = 1,010)**		
Having prescriptions faxed to your pharmacist (so you don’t need to go into the clinic to pick up the prescription)	915	91
Ordering prescriptions online	903	89

^a^The denominator is of people who contacted general practices during the first lockdown

^b^The denominator is people who had or needed a consult during the first lockdown

### Qualitative results

Respondents described where things worked well and where issues occurred with acquiring prescription medication during lockdown. Some issues were specific to or exacerbated by lockdown; others could be relevant at any time.

#### ‘It is incredibly inequitable’: getting a prescription from the practice

At the initial point of requesting a prescription or getting a consultation with a clinician that resulted in a prescription, patients reported diverse experiences. Some reported difficulties in contacting the practice, particularly getting through on the phone to make an appointment or to order a repeat prescription. For those with access to an online patient portal that allowed prescription requests, this was simple and stress-free. Some practices enabled this feature during lockdown, which patients appreciated; others disabled it, forcing people to telephone the practice, which could be time-consuming with long waits to get through.

My GP [General Practitioner] does not usually accept ManageMyHealth [online patient portal] requests so pleased he did for a repeat prescription. (S:F,45-54)

With respect to payment, patients questioned why they were charged an extra fee for an e-prescription, when it was not obvious how this would incur additional costs to the practice. This fee was a disincentive for respondents to request prescriptions online, as it was cheaper to retrieve a physical copy of the prescription from the practice, even though this was inconvenient (and potentially unsafe during a pandemic).

I used a repeat prescription for the first time and was incredibly disappointed in the costs involved... It’s incredibly inequitable and I can understand why people wouldn’t pick up prescriptions or use the service. I mean $24 (non-urgent; $26 urgent same day) to have something faxed that costs $5 to pick up is ridiculously out of balance. I understand that the script involves work from a doctor/nurse before it’s sent, but this is a significant barrier to people being having health needs met in a timely manner. (S:F,25-34)

In recognition of this issue, some general practices waived the ‘fax fee’ during lockdown, reducing the need for physical interactions at the practice.

#### Communication is key: from prescription production to pharmacy

The process of generating the prescription and sending it to the pharmacy could be problematic during lockdown. Delays, sometimes of several days or longer, occurred between the patient ordering a repeat prescription/being issued a new prescription and the script arriving at or being processed by the pharmacy.

At about 4pm today I received a text message from the local pharmacy [to] say that my prescription was now ready to be collected. This is five calendar days, three working days after the doctor sent the script through to the pharmacy. (I:M,65+)

From the patient perspective, it was hard to know whether delays were due to technical or administrative problems with the sending process, delays in the prescription being signed off by a clinician (for repeats), staff forgetting to send the prescription, an overloaded pharmacy, or any combination of these. One respondent made four phone calls to her practice and pharmacy over several days, trying to get her medications, before the problem was identified: an incorrect fax number put into a new fax machine at the practice at the start of lockdown (I:F,35–44). Another respondent reported having to wait for her medicines because the practice only sent ‘groups’ of 10–15 prescriptions to the pharmacy (I:F,45–54), rather than sending each prescription as it was produced. This may have been more efficient for the practice but was inconvenient for the patient.

Several respondents recounted how their prescriptions were sent to the wrong pharmacy, causing confusion and usually multiple contacts between the patient, practice and/or pharmacy. In most cases of error or delay, it was incumbent on the patient to persist with attempts to get the prescription, with practice staff and pharmacies often so busy they responded reactively rather than proactively to problems. Pharmacies that proactively followed up on missing prescriptions provided exemplary service.

My experiences have been on behalf of my elderly (90 s) mother who moved in with us during lockdown. We arranged for 2 monthly renewals of her meds [medications]. In one case, half her meds (medicines prescription) were sent to two different pharmacies; for the next renewal, despite two phone conversations with a nurse from [the general practice], and despite my request to send the script to a specific pharmacy closer to where we live, when I rang the pharmacy I thought we had agreed to use, they had no record of the script…Luckily my mother’s usual pharmacy had her records and so when I rang to see if they had been sent the renewal, even though no script had been received, they offered to follow through with [the practice] and to courier them to us. They phoned back to confirm they had the script and to let me know when to expect the courier and the options for payment. So helpful but I am very concerned that [the practice]’s system was faulty. (S:F,55–64)

When e-prescribing systems worked poorly, some people gave up waiting and picked up a physical copy from the practice to take to their pharmacy, as this gave them control over the process. However, when electronic prescribing systems worked well, patients found the process efficient and expedient.

[It] has been much more convenient. So that [the prescription] went straight to the pharmacy of my choice, electronically…The last time I had to go to the practice, stand in a queue, ask for my prescription...Then go to the chemist, then wait to get my script. It’s a big boon…not to have all that palaver. (I:M,65+)

Some patients reported mistakes in the production of prescriptions, which were mainly logistical. Patients often attributed these to changes in how clinicians were working due to lockdown, e.g. use of telehealth, where the patient did not see the prescription and have the opportunity to point out mistakes (e.g. one respondent was prescribed a medication for anxiety that she was already taking (I: F, 45–54)), or to new and imperfect systems.

I take two restricted medications for a neurobiological condition… The larger workload on my GP practice [in lockdown] meant I spoke to a nurse about my repeat rather than my GP and ended up with only one of my meds because of the nurse’s lack of familiarity with them. It was not life threatening, just inconvenient, and I chose not to call back and get the second medication because even getting the first was such a hassle…It felt easier to go without…than to try and sort it out. (I:F,35–44)

#### Unknowns and uncertainty: prescription processing

At the point of the pharmacy processing the prescription, patients’ experience of delays appeared to vary by pharmacy, depending on how busy the pharmacy was. Although pharmacies were an essential service – and hence could remain open during lockdowns – patients reported that some were closed, or had reduced opening hours during lockdown, which hampered access to prescriptions. Communication to patients about when the prescription was ready also varied. Some patients received a text, or they proactively phoned the pharmacy to check if their medications were ready. When patients were not aware of, or told, how they would be informed of this, or when the communication did not work, it resulted in unnecessary trips to the pharmacy. This could create inconvenience or financial burden if the pharmacy was a distance away, and worry regarding infection, overlaid by the knowledge that police were monitoring compliance with lockdown advice to minimise car trips away from home.

Picked up my prescription but they didn’t have one of the medications which meant I had to go back. I would have liked to know that beforehand so I could make only one trip as I am immunocompromised. (S:F,65+)

#### Heroes and strangers: safely receiving the medication

Delays continued at the point of picking up the medication, with queues and long waits outside the pharmacy because of physical distancing requirements. Some pharmacies provided a home-delivery service, which patients appreciated when it was available, particularly those who were at higher risk of COVID-19. The lack of a delivery option was stressful for those who were uncomfortable with the potential for close physical interaction at the pharmacy or were unable to get there.

My biggest health care heroes have been the pharmacy who drove my scripts over to my house so I wasn’t spending overnight in pain and didn’t put [those in] my ‘bubble’ or self at risk. (S:F,25–34)

As an at-risk person getting medicines from my regular pharmacy has been a huge issue…They won’t deliver and having to get someone on the outside is really awkward on two levels—asking someone to go out of their way to help and…having a stranger (neighbour) see what [medications] you are getting. (S:F,25–34)

Advice and interactions with pharmacy staff at the point of dispensing were highly regarded overall. Patients had very few concerns with safety procedures introduced at pharmacies to reduce the risk of viral spread, such as contactless pickup, one-at-a-time entry systems, physical distancing, Perspex shields at pharmacy counters and hygiene procedures. Although these measures could be intimidating, they were acknowledged to be important and appropriate.

They were really good, they had set up the barrier at the front door, hand sanitiser on the table, eftpos [electronic retail payment system in New Zealand] machine on the table. The first week, probably, everyone was strictly using gloves and masks, not just public but also the staff. (I:F,45–54)

Despite the need for distancing procedures, respondents noticed that these measures could create a lack of privacy as an unintended consequence. Examples were provided about health issues being discussed in full view and hearing of others, without apparent concern for confidentiality.

While waiting in a queue at local pharmacy to pick up medicine, staff asked for personal information and gave instructions loudly in front of other customers in a very indiscreet manner. (S:F,45–54)

#### Explanation needed: changes to medication supply

Once patients had received their medication, lockdown presented other challenges, including the 30-day dispensing limit. It was not clear to respondents whether this dispensing restriction was reasonable and some described negative impacts on themselves and their families. It was also not clear to respondents who should be responsible for informing or helping them with changes in supply, with poor communication about this leading to uncertainty and disruption.

Cannot get my husband’s main medication for his Parkinson’s. Without it he cannot function, work, pay taxes. Nobody can tell me why pharmacy cannot, or will not supply [levodopa]. (S:F,65 +)

Pharmacies not proactively managing medications in short supply. I take one medication that I could only get fortnightly then it disappeared. Felt like the pharmacy could have contacted me in advance of the fortnight ending so I could make a new plan/GPs contact everyone prescribed that medication and offer a switch. (S:F,35–44)

In addition, the risk of exposure to COVID-19 because of dispensing restrictions was not lost on respondents.

They…broke down my 90-day supply into a supply and two repeats, which means of course then I have to go and expose myself to potential infection two more times in order to fill the prescription, which seems…a little counter-productive. (I:M,55–64)

## Discussion

This research explores patient experiences in accessing prescribed medication during lockdown when stringent physical distancing and movement restrictions were in place. Some of the changes to health service delivery during lockdown improved access to medications, but not in all cases. E-prescribing and online prescription requests were convenient for patients when systems worked well, but timely access to prescriptions was made more difficult when systems failed. As also reported internationally, practices and pharmacists were inundated with demand for prescriptions, especially at the beginning of lockdown [39–42], and often provided reactive but not proactive help to address delays or mistakes, leaving the onus on patients to follow these up. Respondents who had bad experiences of e-prescribing preferred the system of physically taking a prescription to the pharmacy, as they knew then how and when they were getting their medication.

### Strengths and limitations

The major limitation of this study was that it involved a self-selecting sample able to complete an online survey. People without internet access or familiarity with digital technology would have been missed, although some respondents reported answering on behalf of family members who would not otherwise have completed the survey. However, if we consider our sample relatively advantaged/digitally literate, their experiences would likely have been better than the general population, so the problems they experienced are probably encountered by many. The survey and interviews covered many other aspects of seeking health care during lockdown, in addition to prescribing and medicines. The description of issues in obtaining prescription medicine was based on qualitative data so we are unable to quantify the proportion of negative experiences and delays compared to interactions that went smoothly. This occurrence is a familiar challenge for those using mixed methods approaches, which aims to use both types of qualitative findings and quantitative data to create deeper understanding of the topic of interest [36, 43]. As a research team, it was important to stay close to the narratives when analysing the interviews, which may lead to differences in the results between to two data sources. It is widely acknowledged that people experienced increased anxiety during lockdown, this may have exacerbated negative interpretation of events that occurred at that time [44]. In addition, just as in the survey, self-selection into the interviews may have led to a different type of selection bias, with those having negative experiences with accessing health care nominating themselves for an interview; nonetheless, 436 respondents volunteered to be interviewed and we purposely selected people from this list further minimising the opportunity for interviewing those who had negative experiences.

This paper focused on the *patient* experience; research from the prescriber and pharmacist perspectives would complement these findings and help elucidate reasons for barriers that patients encountered, which may include information technology integration issues and pharmacy’s lack of access to patient information [45].

Although this research provides the NZ experience, health systems globally are experiencing transformation and hurried change due to COVID-19, including e-prescribing changes [14]. Hence, our findings are likely to be relevant to other jurisdictions.

### Comparison with existing literature

One service that proved useful in the lockdown context for the participants in this study was home delivery of prescription medicines, particularly for vulnerable patients (also see Table 5). Only a small proportion of respondents experienced this service, whereas in other countries, home delivery was rapidly introduced during the pandemic. For example, in Cape Town, home delivery was instigated to reduce the exposure of people with chronic conditions to COVID-19 [46] and home delivery was part of a suite of remote pharmacy services in China [47]. In the UK, reimbursement to pharmacists was introduced for home delivery of medicines to vulnerable patients with COVID-19 and those self-isolating [48], but demand for this service was so high that volunteers were sought to undertake deliveries [49].

Although there was a huge increase in adoption of NZePS during lockdown by general practices, data from August 2020 showed a dip in e-prescriptions issued, from a peak in July 2020 [12]. The reasons for this decline are unknown, but other research has found that transitioning to e-prescribing systems takes time, commitment, ongoing training and efficient systems [50] and lack of provider support and system errors can be significant barriers [51]. NZ prescribers may need additional support to maintain their use of NZePS.

Despite some issues with obtaining prescriptions during lockdown, respondents wanted online ordering and e-prescriptions to continue post-lockdown, suggesting the process overall was satisfactory. This is consistent with the generally positive reception of e-prescribing from patients internationally, particularly for improvements in convenience and safety [22, 52–54].

### Implications for research and/or practice

Prescription costs (including indirect costs, e.g. travel time) can exacerbate inequities in access to medicines [55], especially for disadvantaged groups [56, 57]. In this sample, the cost of an e-prescription varied. E-prescribing could be expected to reduce workload, as practices do not have to post/fax hard-copy prescriptions to pharmacies, and pharmacies do not have to spend time chasing these up [18, 58], so more transparency in additional charges for e-prescriptions is needed.

From patient experiences in this research, systems need better checks to ensure correct medications are prescribed, prescriptions are sent to the correct pharmacy, and errors can be detected and corrected through effective communication between practices and pharmacies. Such learning could be assisted by adoption of an e-prescribing incident reporting tool [59], especially if patients were also enabled to report. Secure e-prescribing systems should perform better than outdated systems such as fax which has well-documented security and inefficiency issues [60], so much so, that health services in the UK and NZ were instructed to remove and replace all fax machines in 2020 [61, 62].

Another area for improvement is better communication with patients about how new prescribing systems are expected to function, including during a pandemic. The introduction of e-prescribing generally requires more engagement with, and information for, patients [22, 52–54]. Better communication with patients affected by drug shortages likely requires a multipronged approach by both general practices and community pharmacies, who receive and transmit messages from the agencies responsible for purchasing and monitoring drug supply to patients, but then also follow-up by identifying alternative treatments. Communication with patients is especially important when systems are changing; ensuring that communication from prescribers and pharmacies about the process of (and options for) access to prescriptions, delivery and collection of medications and costs is important for patients.

## Conclusion

Changes to service delivery are typically time-consuming and slow. The COVID-19 pandemic sped up change processes, effectively side-stepping usual elements of resistance, such as system readiness, adopter characteristics and implementation obstacles [63]. This research suggests that now is the time to lock-in changes such as e-prescribing that have improved patients’ access to prescriptions, and to address barriers such as cost and unclear communication with patients. The patient perspective is necessary in the co-design and evaluation of such systems.

## Supplementary Information

**Additional file 1.** Include interview schedule and survey questionnaire.

## Data Availability

The datasets used and/or analysed during the current study are available from the corresponding author on reasonable request.
