# Supplementary material for: E-prescribing and access to prescription medicines during lockdown: experience of patients in Aotearoa/New Zealand
Source: BMC Fam Pract. 2021 Jul 1;22:140. doi: 10.1186/s12875-021-01490-0 (PMC8247618; doi:10.1186/s12875-021-01490-0)
Supplement: Supplementary file 1 — Additional file 1. Include interview schedule and survey questionnaire. [file 12875_2021_1490_MOESM1_ESM.zip › Interview schedule - patients experiences of health care in a pandemic_FINAL.docx]

E-prescribing and access to prescription medicines during lockdown: experience of patients in Aotearoa/New Zealand

Authors:

Fiona Imlach^[[1]](#footnote-1)^, Eileen McKinlay^[[2]](#footnote-2)^, Jonathan Kennedy^2^, Caroline Morris^2^, Megan Pledger^1^, Jacqueline Cumming^1^, Karen McBride-Henry^[[3]](#footnote-3)^, Corresponding author: [karen.mcbride-henry@vuw.ac.nz](mailto:karen.mcbride-henry@vuw.ac.nz)


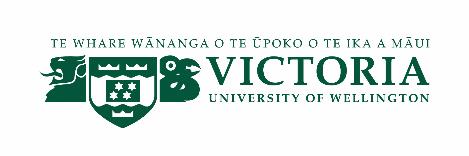


**Patient experiences of primary health care in a pandemic**

**INTERVIEW SCHEDULE**

Thank you again for agreeing to be interviewed for this research which focuses on people’s experiences of general practice care during the COVID-19 pandemic lockdown period.

Living in general has been very difficult for many during the pandemic and I want you to know that we can stop this interview at any point if you are feeling uncomfortable for any reason. Let me know if you need to take a break.

Do you have any questions about the research before I begin?

**First some general questions about yourself and your wellbeing and health during COVID-19.**

1. How have you found being in lockdown?
2. Who was in your lockdown bubble?
3. How concerned are you about the impact of the lockdown or COVID-19 on your health?

**Now, some questions about the (general practice/GP clinic/health centre etc) you go to.**

1. What is the general practice or health centre or clinic that you regularly attend? Do you have a doctor there that you usually see?
2. Do you know if [this practice] call themselves a Health Care Home?

**Moving onto some questions about your health care during the COVID-19 pandemic.**

1. During the COVID-19 pandemic lockdown, did you have to seek health care or advice from your GP clinic/health care centre? Tell us about this. *[If they haven’t, go to question 7 about delay]*
   Some prompts:
   1. Did you contact your regular general practice or another service? Did you see or talk to a clinician you already knew or someone unfamiliar? How important to you was contacting someone you knew in this circumstance?
   2. How easy (or not) was it to get in contact?
   3. Generally speaking, what did you seek advice or care about? (e.g. COVID, screening, acute illness, chronic illness, care for another person, immunisation)
   4. Who did you seek advice or care from at the centre e.g. a doctor or nurse or nurse practitioner?
   5. What type of contact did you have e.g. a physical visit/phone call/video call/e-portal consultation or something else
   6. For telephone, virtual or other new methods: Was the method of contact new to you? How did you find the consult – what was good and not so good about it? Would you like to use this method when things settle down?
   7. For face-to-face visits: how was this different from a usual face-to-face visit before the pandemic? What was good and not so good about it? How did you access the visit e.g. phoning the practice first, having some kind of triage, going through the receptionist. What was this like? E.g. was the triage experience new, did it work, how did they feel about it especially depending on who did it.
   8. What was the outcome of the interaction, did you get what you needed?
   9. Did you pay for the care and information? How much was it (if you want to tell us that) – or was this more or less than a usual consult?
   10. Did you have to get a prescription, and how did that happen? Was this different than usual?
   11. [Speculative question, to ask everyone] Non face-to-face consult methods such as telephone or video consults may work better for some people than others – and better for some health problems than others. In what circumstances do you think non face-to-face consults would work well for you, and when do you think they wouldn’t work so well?
2. During the COVID-19 pandemic lockdown, did you delay or put off getting help for a health issue that you would have got help for under usual circumstances? Tell us about this. Some prompts:
   1. What did you delay or put off?
   2. Why did you delay or put this off? Barriers may be cost, fear of infection, thinking that health care workers were too busy, etc.
   3. What could have been done so that you didn’t delay?
   4. When will you get this health issue addressed?
   5. How does this delay make you feel?
3. During the COVID-19 pandemic did you make any changes to how you usually manage your health or care for yourself? These could be positive or negative changes. For example changes to diet, exercise, taking (or not taking) medicines, taking alcohol or drugs; managing isolation and stress; accessing other health services such as pharmacists. If yes:
   1. What changes did you make and why?
   2. What difference have these changes made? Will you continue them after the lockdown?

**Questions for those with long-term conditions**

1. We know that people with chronic or long-term health condition often experience some difficulties in daily life and may need to visit their general practice more often. Do you have one or more chronic health conditions? (prompt if needed, e.g. heart disease, diabetes, high blood pressure, depression, anxiety, gout, arthritis, asthma, respiratory disease, etc). If yes, explore with the following prompts. If no, go to the final questions.
   1. Before COVID-19, how did your general practice help you manage your health?
   2. Did this/has this changed during the COVID-19 pandemic? Prompts:
   3. Did staff from your regular general practice contact you to check on how you were getting along?
   4. Were you able to get a flu vaccination if you wanted one? How did this work for you?
   5. How did things go with getting repeats of your medicines?
   6. Were you able to go to the pharmacy to pick these up?
   7. Do you normally get care from a physiotherapist or other health or social care professional? If yes, how did you access this during the pandemic?

**Final questions**

1. Is there anything else you want to tell us about your health and health care during the pandemic? Is there anything that could have been done differently to make your experience of health care better – e.g. better communication, better access to health care, better information about what services were available?

**Demographics**

Can I ask you a few questions about yourself.

1. Which age group do you belong to:

- 18-24
- 25-34
- 35-44
- 45-54
- 55-64
- 65-74
- 75-84
- 85 or older

1. Can you tell me which ethnic group or groups you belong to?

- New Zealand European
- Māori
- Samoan
- Cook Islands Māori
- Tongan
- Niuean
- Chinese
- Indian
- Another ethnic group such as Dutch, Japanese or Tokelauan? Please say what it is___________________________________________________________________

1. Which of these statements best describes your **current** work situation (during the lockdown):
   - In paid employment (including self-employment) as before coronavirus
   - In paid employment (including self-employment) but with reduced pay due to coronavirus
   - In employment but not being paid because of coronavirus
   - Not in paid employment and not looking for a job (for any reason, such as being retired, a caregiver, a homemaker, a full-time student)
   - Unemployed (not due to coronavirus) and looking for a job (now or after lockdown)
   - Unemployed as a direct result of coronavirus and looking for a job (now or after lockdown)
   - Other (please specify)
2. Where do you live?
   - Northland
   - Auckland
   - Waikato
   - Bay of Plenty
   - Gisborne
   - Hawke’s Bay
   - Taranaki
   - Manawatu-Whanganui
   - Wellington-Wairarapa
   - Tasman
   - Nelson
   - Marlborough
   - West Coast
   - Canterbury
   - Otago
   - Southland
   - Other (specify)
3. What is your gender? (If not already evident from the interview)
   - Male
   - Female
   - Gender diverse
   - Prefer not to say

We will send you a $50 grocery voucher once lockdown has ended to thank you for your time – can you give me your postal address for that? Do you have a preference for which supermarket?

**Support resources if patients become distressed**

Healthline 0800 611 116

COVID Healthline 0800 358 5453

General mental health support

<https://www.depression.org.nz/>

<https://www.allright.org.nz/>

<https://www.thelowdown.co.nz/>

<https://www.auntydee.co.nz/tips-and-help> (mental health resource for Pasifika)

1. Health Services Research Centre, Victoria University of Wellington, New Zealand [↑](#footnote-ref-1)
2. Department of Primary Health Care and General Practice, University of Otago Wellington, New Zealand [↑](#footnote-ref-2)
3. School of Nursing, Midwifery, and Health Practice, Victoria University of Wellington, New Zealand [↑](#footnote-ref-3)
